# A New Caffeine Detection Method Using a Highly Multiplexed Smartphone-Based Spectrometer

**DOI:** 10.3390/bios14120590

**Published:** 2024-12-03

**Authors:** Erhuan Zhuo, Huanxin Xia, Huan Hu, Yu Lin

**Affiliations:** Zhejiang University-University of Illinois Urbana-Champaign Institute, Zhejiang University, Haining 314400, China; 22271102@zju.edu.cn (E.Z.); huanxinxia@intl.zju.edu.cn (H.X.); huanhu@intl.zju.edu.cn (H.H.)

**Keywords:** aspirin, caffeine, on-site detection, point-of-care testing, smartphone-based spectroscopy

## Abstract

Smartphones equipped with highly integrated sensors are increasingly being recognized as powerful tools for rapid on-site testing. Here, we propose a low-cost, portable, and highly multiplexed smartphone-based spectrometer capable of collecting three types of spectra—transmission, reflection, and fluorescence—by simply replacing the optical fiber attached to the housing. Spectral analysis is performed directly on the smartphone using a custom-developed app. Furthermore, we introduce a high signal-to-noise ratio (SNR) caffeine detection scheme that leverages aspirin and salicylic acid as fluorescent probes, allowing for the rapid and straightforward detection of caffeine in various samples. The fluorescence quenching of the probes was found to be linearly related to the caffeine concentration (0–200 μM), and the recoveries of the commercially available caffeine-containing samples were in the range of 98.0333–105.6000%, with a limit of detection (LOD) of 2.58 μM. The reliability and stability of the on-site assay using the smartphone spectrometer were verified. More importantly, this spectrometer demonstrates great potential as a versatile device for use outside of laboratory settings by enabling different operating modes tailored to various scenarios.

## 1. Introduction

Spectral analysis is extensively utilized in biochemical testing [1], medical diagnostics [2], environmental protection [3], and food safety [4]. It serves as a reliable method for both visualization and detection. Although commercial spectrometers offer high accuracy and resolution, their bulkiness and complexity often pose limitations when conducting on-site testing. Meanwhile, the high cost of these devices restricts their widespread use [5]. Since the early 21st century, smartphones have emerged as highly popular electronic devices, offering fast information processing, large storage capacity, and unparalleled mobility. Smartphones come equipped with components such as complementary metal oxide semiconductor (CMOS) cameras with millions of pixels, LED flashlights, graphical processing interfaces, and customizable applications. These features not only provide essential optical components but also function as a platform for data processing, enabling their application in optical signal analysis [6,7,8,9,10].

In contemporary research of smartphone-based spectroscopic analysis, lenses and optical fibers are primarily used to guide and focus light onto a diffraction grating at a specific angle, allowing the primary diffracted light to be captured by a CMOS sensor. According to grating theory, the dispersion on the plane of the CMOS sensor can be approximated as linear. Therefore, the relationship between pixel position and wavelength can be established through RGB images captured by the smartphone’s camera. This is obtained using light sources of known wavelength, such as lasers or monochromatic LEDs, or by analyzing the chromaticity or hue of images [11,12,13,14,15,16]. The intensity values corresponding to the same wavelength are then summed to obtain the overall intensity for that wavelength.

These methods effectively translate the color information captured by the smartphone into the wavelength-intensity profile necessary for spectral analysis. Additionally, Ding et al. [17] proposed a combination of wavelength and intensity calibration to further address the issue of nonlinear sensitivity in smartphone sensors, which improves spectral accuracy. In this paper, we emphasize the use of image processing techniques to further improve this accuracy, as will be discussed in subsequent sections.

Grating-based smartphone spectrometers are widely employed to analyze transmission [18,19,20,21], fluorescence [11,16,22], and reflectance spectra [23,24] and have been successfully applied in the fields of bioassay, agricultural development, medical diagnostics, food and drug safety, and environmental protection. However, most of the early-stage smartphone-based spectrometers were limited to analyzing a single type of spectrum, which clearly hinders smartphone spectrometers from bringing convenience to more scenarios. To enable the detection of different samples using the same smartphone spectrometer, researchers have explored the multiplexing of optical paths in these devices. For example, in the work of Xiao et al. [22], transmission and fluorescence spectroscopy were accomplished by arranging light sources in different directions of the cuvette. This approach has broadened the range of the testing samples with a compact smartphone-based spectrometer. Additionally, Kenneth D. Long et al. designed a smartphone spectrometer that analyzes absorption, fluorescence, and photonic crystal (PC)-based label-free detection based on a transmission spectrometer [20,25]. In addition, they proposed an independent but complementary optical path combined with a cartridge consisting of a series of linear liquid compartments, accomplishing the measurement of three different types of spectra [26]. These innovations have expanded the range of samples by multiplexing the light paths, thereby centralizing the entire system. As summarized in [27], different types of spectra have their detection advantages on different target samples.

**Figure 1:**
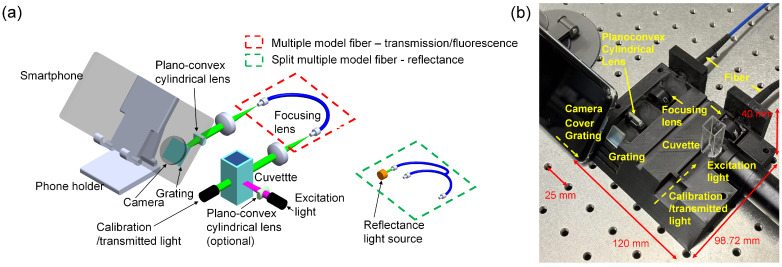
Light path and fabrication of the smartphone-based spectrometer. (**a**) Light path and fabrication of the spectrometer. (**b**) Smartphone spectrometer physical picture.

**Figure 2:**
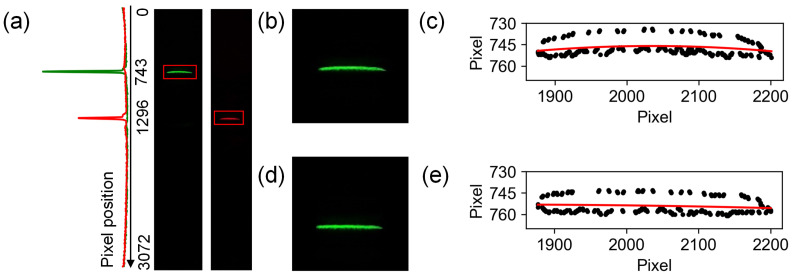
Spectrum RGB images processing. (**a**) RGB ROI (red boxes) and peak pixel position of 532 nm and 650 nm. Resolution = (650 − 532)/(1296 − 743) ≈ 0.21 nm/pixel. (**b**–**e**) Spot and contours of 532 nm RGB image before and after correcting.

In this paper, we successfully developed a highly multiplexed smartphone-based spectrometer, as shown in Figure 1, and employed it to detect caffeine in commercially available drinks. The spectrometer is capable of measuring and analyzing three types of spectra in the visible range (350–750 nm) with a resolution of 0.21 nm/pixel, as shown in Figure 2a. The device consists of a compact 3D-printed shell measuring only 120 mm × 40 mm × 107.3 mm, along with a grating, optical fibers, lenses, and a smartphone. Image processing is performed through a customized app on the smartphone, which visualizes the data as a spectral curve, generates a standard curve, and displays measurement results. By analyzing the spectra of Rhodamine dye (R6G) and skimmed milk, we verified that the developed smartphone spectrometer achieves a high degree of multiplexing of the optical path while accurately measuring the spectrum. Additionally, we present a high SNR scheme for detecting caffeine in beverages using aspirin and its hydrolysis product, salicylic acid, as fluorescent probes. This method demonstrated that the fluorescence quenching of aspirin and salicylic acid was highly correlated with the caffeine concentration, successfully overcoming the challenges of rapid hydrolysis and weak fluorescence of aspirin in determining caffeine levels using the smartphone-based spectrometer. The LOD of this scheme was 2.58 μM, enabling a rapid, sensitive, and convenient fluorescence method outside the laboratory. Considering the advantages of resolution, cost, structure, analytical speed, and accuracy, we believe the proposed smartphone-based spectrometer can be widely adopted for on-site detection.

## 2. Materials and Methods

### 2.1. Optical Design and Fabrication of Smartphone-Based Spectrometer

The smartphone-based spectrometer primarily consists of three parts: the smartphone and its holder, the housing, and the optical components, as depicted in Figure 1.

(1) The smartphone and its holder: The holder allows for quick attachment and easy adjustment of the smartphone’s position relative to the housing. The HUAWEI P40 Pro smartphone (Huawei Technologies Co., Ltd., P40 Pro, Shenzhen, China) is used, featuring a professional camera mode that allows the adjustment of various parameters, including exposure time *S* (S=1/10 for calibration, transmission modes; S=1/2 for reflectance mode; S=1 for fluorescence mode), sensitivity ISO (ISO=50 for calibration, transmission modes; ISO=1600 for reflectance mode; ISO=1000 for fluorescence mode), white balance mode (WB = ‘Cloud’), and focal length (f=∞). These settings enable the capture of 4096 × 3072 pixel RGB images.

(2) Housing: The housing was designed using SolidWorks 2021 and produced using PLA material on an Ender-3s13D printer (Shenzhen Creality 3D Technology Co., Ltd., Shenzhen, China). The housing is split into two parts that are screwed together, with a small size of 120 mm × 40 mm × 107.3 mm. Black tape is used to cover any potential light leakage gaps. Given that the smartphone camera module often protrudes from the rear surface, a notch matching the camera module’s size was designed on the housing’s beveled surface. This design helps secure the phone’s position in conjunction with the holder.

(3) Optical components: These include two plano-convex cylindrical lenses (CY104109, f = 20 mm; LBTEK, Changsha, China), two biconvex lenses (MBCX10303, f = 15 mm; LBTEK, China), diffraction grating (GT13-12, 1200 Grooves/mm; Thorlab, Shanghai, China), multimode optical fiber and flange (MMC200L-0.37-PC-1, 300–2400 nm; LBTEK, Changsha, China), and split-multimode optical fiber (SPLY200-2-VIS-NIR, 400–2200 nm; HangzhouSPL Photonics Co., Ltd., Hangzhou, China) directly mounted into the 3D printed housing. Two laser pointers (DL55202, λ=532 nm; DL55200, λ=650 nm; Deli, Ningbo, China) were used for calibration, and the 532 nm laser pointer could also be used as an excitation source for fluorescence spectroscopy. UV LED (UVGO; λ=275 nm, Zhongshan Guzhen Youweigu Lighting Appliance Factory, Zhongshan, China). A smartphone (iPhone 15; Apple Inc., California, CA, USA) flash was used as a light source for the transmission spectrum.

Different operating modes can be enabled by turning on different light sources. Both the calibration and transmission light pass directly through the cuvette, and the reflected light passes through the reflective surface and exits from the cuvette. Fluorescence is excited by an excitation light source perpendicular to the direction of the light path, avoiding the collection of excitation light. A lens focuses the light transmitted through the cuvette onto the incident surface of the optical fiber. This light is then transmitted from the outlet surface and converged into a linear spot on the grating after shaping by the converging and cylindrical lenses. The angle between the grating and the light path is set to 47°, allowing the first-order diffracted light to enter the mobile phone camera. Using a linear interpolation approach, the resolution in the spectral direction was calculated to be 0.21 nm/pixel based on the pixel scale and the calibration wavelength.

### 2.2. Image Processing

The primary working principle of the smartphone-based spectrometer involves processing the spectral RGB images captured by the smartphone camera to derive the wavelength-intensity curve. Initially, the pixel–wavelength calibration is performed using laser pointers with 532 nm and 650 nm wavelengths. The laser spot images require further processing of the intensity values (*V*) of individual pixels. Optional processing includes using the average intensity value of RGB channels as (1) or a weighted average value of the RGB channels as (2), which is used in consideration of human color perception, as the intensity, *V*, of each pixel [28]. By analyzing the RGB images of these lasers, the region of interest (ROI), as shown in Figure 2a, and the pixels where the wavelengths were located is identified according to the intensity value calculated from (2).

(1) V=(R+G+B)/3

(2) V=0.299∗R+0.587∗G+0.114∗B

When the grating is positioned close to the phone camera, the relationship between the wavelength and pixel position of spectra images is approximately linear, allowing the pixel–wavelength relationship to be established through linear interpolation. Therefore, the intensity is summed in the non-spectral direction to yield intensity values for the corresponding wavelengths. Ultimately, the wavelength-intensity curve is plotted along the spectral direction.

However, light passing through the convex and cylindrical lenses comes with spherical aberration, and the line spot in the ideal case is bent (Figure 2b). To improve calibration accuracy, the images were corrected based on the quadratic curve of contour fitting for the 532 nm calibration light (Figure 2c–e). The primary cause of this bending is spherical aberration, which varies with wavelength. Correcting the image using this wavelength is reasonable, considering that the 532 nm calibration light is located in the middle of the visible spectrum.

### 2.3. Experimental Reagents and Instrument

Rhodamine dye (R6G) (≥98%) was purchased from Aladdin Bio-Chem Technology Co., Ltd. (Shanghai, China). Caffeine (≥98%) was purchased from Chengdu Must Bio-technology Co., Ltd. (Chengdu, China). Aspirin (≥98%) was purchased from Goad Laboratory Practice Bioscience (Shanghai, China). Glacial acetic acid (≥99.5%) was purchased from Wuxi Jingke Chemical Co. (Wuxi, China). All reagents were experimentally pure and did not require further purification. Skimmed milk, jasmine tea (Sample 1), Red Bull (Sample 2), Monster Energy (Sample 3), self-ground coffee (Sample 4), and Luckin Americano (Sample 5) were purchased from the local market for analysis of caffeine content. Pure water was purchased from Hangzhou Wahaha Beverage Co. (Hangzhou, China). The analytical balance was purchased from Changzhou Lucky Electronic Equipment Co., Ltd. (JA203H; Changzhou, China).

### 2.4. Sample Preparation

Rhodamine solutions of 0–10 μg/mL were obtained using R6G powder dissolved in pure water. Different dilution ratios of skimmed milk were prepared using skimmed milk and pure water. The mixed solution of caffeine and aspirin was obtained in the following steps and used to establish the standard curve.

- Aspirin solution: Take 10 mg aspirin and dissolve it in 20 mL pure water to obtain 500 ppm aspirin.
- Caffeine solution: Take 15 mg caffeine and dissolve in 75 mL pure water, diluted to obtain 400 μM and 200 μM caffeine.
- Take part of the aspirin solution and add glacial acetic acid at 1% volume fraction of the aspirin solution (to inhibit hydrolysis).
- Prepare the mixed solution of aspirin (50 ppm) and caffeine (0–200 μM) in every cuvette using the above solution.
- Transfer the cuvette with the mixed solution to the smartphone-based spectrometer and collect the fluorescence spectrum for analysis.

For the actual sample testing, five commercially available beverages from the previous subsection (S1–S5) were quantified for caffeine. The five samples were diluted with pure water and transferred to the cuvette. The volume of each solution in the cuvette was adjusted to 2.5 mL.

## 3. Results and Discussion

### 3.1. Validation of Spectrometer

**Figure 3:**
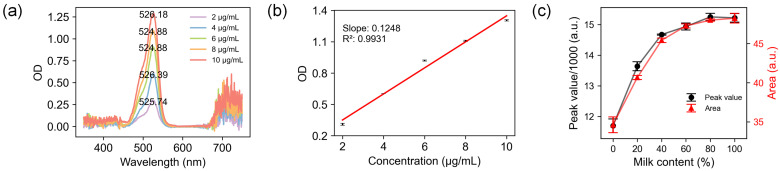
Validation of spectrometer using R6G. (**a**) Absorption spectrum of R6G. (**b**) Linear fit of OD value and concentration of R6G. (**c**) 650 nm reflective intensity of diluted milk. Milk content in diluted solution is 0, 20, 40, 60, 80, 100%.

To validate the optical path and image processing workflow of the smartphone-based spectrometer, R6G solution and skimmed milk were employed as test objects. R6G is a widely used fluorescent dye in laser technology, fluorescence microscopy, biomarkers, and other optical experiments [29]. Typically, R6G appears as a dark-orange powder and exhibits strong absorption around 526 nm when dissolved in water. The transmission spectra of pure water and a series of aqueous R6G solutions were processed and analyzed to extract the absorption spectrum of the R6G. The absorption spectra revealed peak positions near 526 nm across solutions in five different concentrations (Figure 3a). In the test of homogeneity of OD values, the *p*-value of Levene’s tests reached 0.6429 (>0.05), indicating that variance is homogeneous. Further analysis of the optical density ($OD\;=\;-\log_{10}\left(T_{\mathrm{water}}/T_{R6G}\right)$) of peaks near 526 nm revealed that, at low concentrations (2–10 μg/mL), the OD of R6G solutions showed a strong linear relationship with its concentration (Figure 3b). The analysis of variance (ANOVA) reports showed a *p*-value of $6.86434*10^{-5}$, which indicated that the regression equation explained the data effectively and that the linearity is consistent with the Lambert–Beer Law. The signal light measured in transmission and fluorescence mode from the cuvette follows the same optical path. To further verify the operation of the device in reflectance mode, the 650 nm laser pointer was placed at the *Reflectance light source*, as shown in Figure 1a, and skimmed milk diluted in different ratios was added to the cuvette. The measured intensity corresponds to the peak value of the reflectance spectrum around 650 nm and the area below the 645–655 nm curve. The results showed that the reflected light intensity could correctly indicate the dilution ratio of the purchased skimmed milk (Figure 3c).

These validations confirm that the smartphone spectrometer’s optical path can effectively collect transmission, fluorescence, and reflectance light, and the corresponding image processing procedures can accurately perform quantitative measurements of spectral data.

### 3.2. Application of the Smartphone-Based Spectrometer in the Detection of Caffeine

**Figure 4:**
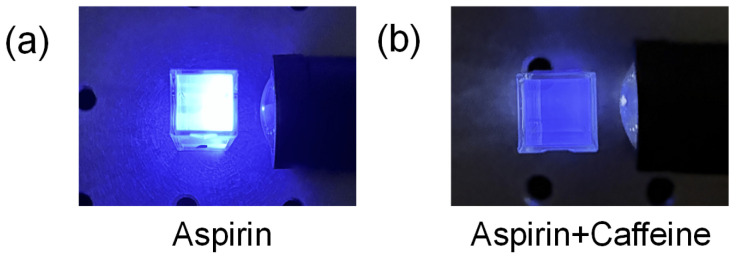
(**a**) Aspirin fluorescence excited by UV LED. (**b**) Aspirin fluorescence is quenched by caffeine.

Following the validation of the smartphone-based spectrometer’s accuracy and stability, the device was subsequently employed for the rapid detection of caffeine using aspirin in fluorescence mode. Caffeine is a xanthine alkaloid compound that is safe for adults to consume less than 100 mg per day. Most previous caffeine testing methods have been time-consuming and expensive, highlighting the need for more efficient approaches. Aspirin, a common component in pain relievers, antipyretics, and anti-inflammatory drugs, relieves mild or moderate pain, reduces fever, and prevents blood clots. Smith et al. have shown that caffeine suppresses the fluorescence of aspirin [30]. This quenching effect is demonstrated in Figure 4.

Aspirin is slightly soluble in water and quickly hydrolyzed to salicylic acid and acetic acid as (3). An aqueous aspirin solution emits a weak light-blue fluorescence in the 400–450 nm range when excited by 275 nm UV light. This quenching effect primarily arises from forming a complex between caffeine and aspirin through π−π stacking interactions between their aromatic rings. However, this method necessitates that the operator complete the experiment promptly due to the rapid hydrolysis of aspirin and its slow dissolution in water.
(3)
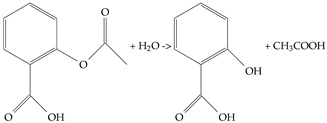


Here, we demonstrate a novel caffeine detection method utilizing aspirin with a smartphone spectrometer, effectively addressing the issues related to aspirin’s dissolution rate and hydrolysis. Furthermore, applying a smartphone spectrometer for fluorescence collection circumvents the constraints of traditional laboratory equipment in this assay.

#### 3.2.1. Reactions of Caffeine and Aspirin

To investigate the influence of hydrolysis on the fluorescence of aspirin during its reaction with caffeine, acetic acid was introduced into a mixed solution containing varying concentrations of caffeine and aspirin. The introduction of acetic acid inhibits aspirin hydrolysis, enabling the study of aspirin’s hydrolytic behavior and its impact on caffeine detection protocols.

**Figure 5:**
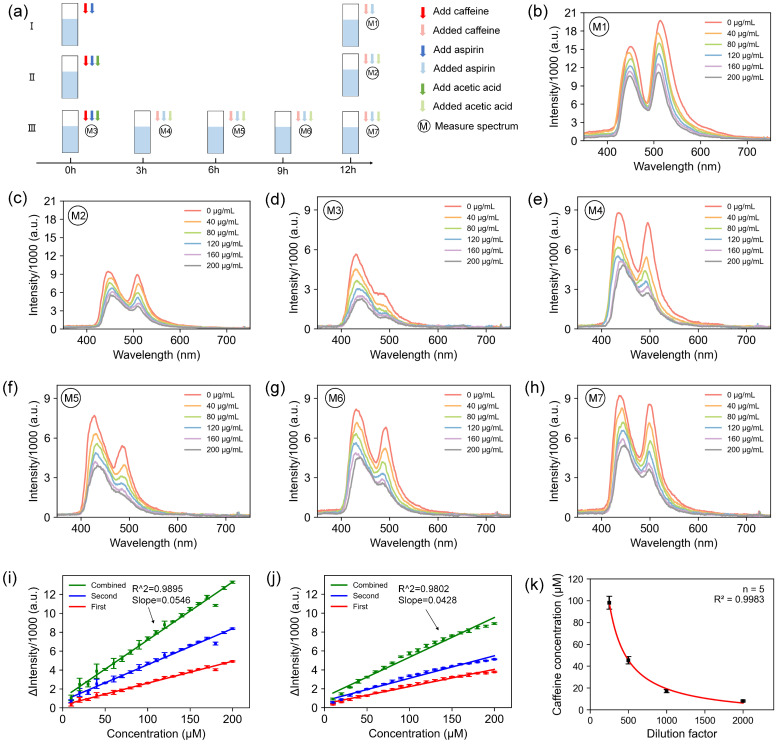
Effect of the presence or absence of inhibition of aspirin hydrolysis on its fluorescence. (**a**) Experiment design under group I, II, III. M1–M7 are the specific cases of measuring spectrum. (**b**,**c**) Fluorescence spectra after 12 h reaction with caffeine under inhibited and uninhibited conditions. (**d**–**h**) Fluorescence spectrum obtained by hydrolyzing 0, 3, 6, 9, 12 h aspirin with caffeine. (**i**,**j**) Linear fit results of quenching fluorescence and caffeine concentration in Figure 5 M1 and M2. (**k**) Caffeine concentration in coffee diluted at different times. The red line is the result of fitting dilution factor and caffeine concentration.

Figure 5a(I/II) shows the fluorescence spectra were measured after 12 h. The corresponding results in Figure 5b,c demonstrate that the sample lacking glacial acetic acid exhibited stronger fluorescence compared to the sample containing acetic acid. The hydrolysis proceeds with a change in the concentration of the fluorescent probes and the production of acetic acid, which alters the solution pH, gradually increasing the fluorescence intensity. Meanwhile, the double peaks in the fluorescence spectra suggest the presence of another fluorescent probe other than aspirin in the mixed solution, whose fluorescence was similarly quenched by caffeine. Sequential measurements of fluorescence spectra at 3 h intervals (Figure 5a(III)) were then implemented. The result spectra showed a consistent presence of the first peak, with the second peak gradually intensifying (Figure 5d–h). Since acetic acid does not have any fluorescent properties in the visible range, the findings confirm the source of each peak; the first peak corresponds to fluorescence from aspirin, while the second peak originates from its hydrolysis product, salicylic acid.

The spectra reveal that varying caffeine concentrations significantly quench the fluorescence of both aspirin and salicylic acid. The linear fit results of caffeine concentrations versus fluorescence quenching at the first peak (shorter wavelength), the second peak (longer wavelength), and the sum of them (*combined*) all demonstrated a clear linear relationship (Figure 5i,j). The *p*-value of total quenching intensity reaches 0.9959 and 0.9997 in the case of Figure 5a M1 and M2, respectively, showing the homogeneous variance of the data. The *combined* lines in Figure 5i,j indicate a strong linear correlation under both uninhibited and inhibited aspirin hydrolysis conditions ($R^{2}\;=\;0.9895$ and $R^{2}\;=\;0.9802$, respectively) with the *p*-value of $4.5329*10^{-26}$ and $2.0507*10^{-17}$ of ANOVA tests showing that the regression equations explain the data effectively. However, the SNR and the linear fit are notably better when aspirin hydrolysis is not inhibited. Therefore, we first chose the 24 h-hydrolyzed aspirin and caffeine to obtain a standard curve similar to the *combine* one in Figure 5i to investigate the caffeine content of self-ground coffee. The caffeine concentration in each diluted sample was determined using the established standard curve, showing a direct relationship with the dilution factor. The variance in the determined caffeine concentration decreased as the caffeine concentration was reduced (Figure 5k), indicating the importance of proper sample dilution.

**Figure 6:**
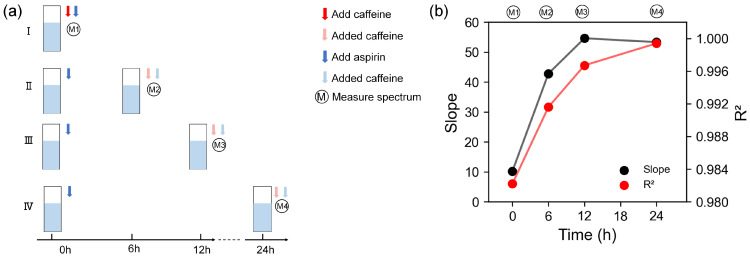
The effect of the degree of aspirin hydrolysis on the linear fitting results when aspirin hydrolysis is not inhibited. (**a**) Experiment design of the effect of aspirin hydrolysis time under under group I, II, III, IV. (**b**) Linear fit results of different aspirin hydrolysis times.

To further assess the impact of the degree of aspirin hydrolysis on the caffeine detection performance, experiments were conducted at various hydrolysis times (0, 6, 12, and 24 h), as shown in Figure 6a. Fluorescence intensity measurements were performed after 20 min. The cumulative fluorescence quenching of the two fluorescent probes was measured at different caffeine concentrations and was then linearly fitted to the caffeine concentration, similar to the *combined* lines in Figure 5i. The results indicated that hydrolysis enhanced the effectiveness of aqueous aspirin solutions in establishing standard curves, with optimal stabilization and LOD (LOD=$\frac{3\sigma }{K}$, σ-standard deviation of the blank, *K*-slope of the standard curve) observed after 12 h of hydrolysis (Figure 6).

#### 3.2.2. Real Sample Testing

Based on the aforementioned experiments, the reaction between 24 h-hydrolyzed aspirin and caffeine was investigated, and a standard curve was established by fitting a straight line to the total fluorescence bimodal quenching and caffeine concentration after 20 min. The caffeine content in real samples can be determined with unknown concentrations by first diluting to the working range (0–200 μM), followed by measuring the fluorescence quenching in mixed solution with the established standard curve.

**Figure 7:**
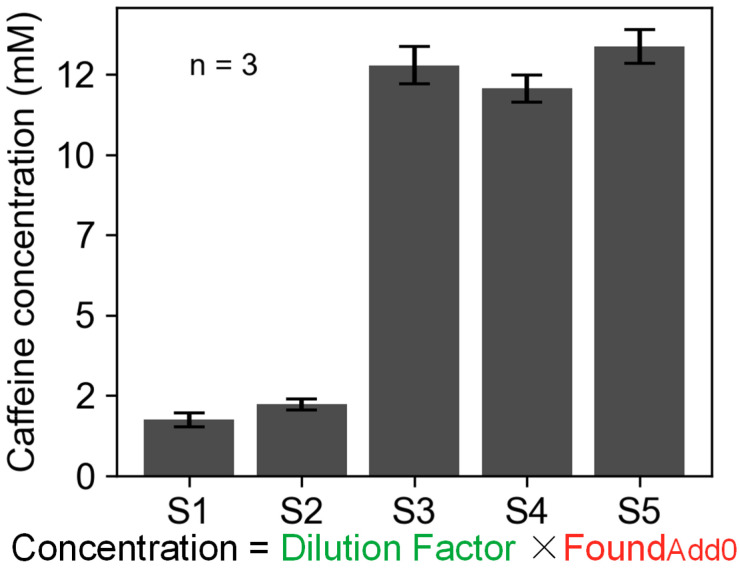
The original caffeine content in five samples. Data for red and green flags are from Table 1.

For commercially available caffeine-containing beverages, samples S1–S5 were diluted to fall within the linear working range of the smartphone-based spectrometer’s caffeine concentration measurement. A 10 μM standard caffeine solution was added to each diluted sample, and the caffeine content before and after the addition was determined based on the fluorescence intensity. The recovery of each sample was calculated (Table 1). The five samples, including tea beverages, energy drinks, and coffee, demonstrated recoveries ranging from 98.03 to 105.60%. Then, the original caffeine concentration in the undiluted samples was calculated by analyzing the caffeine content prior to standard addition and accounting for sample dilution (Figure 7). These results confirm that this caffeine detection scheme is reliable for accurately determining caffeine content in commercially available beverages, which can be accomplished by simply mixing common stable solutions. Although the smartphone-based spectrometer detection accuracy is lower than that of other laboratory equipment, the proposed device is more suitable for on-site testing due to its compact size and lower cost.

This smartphone spectrometer offers impressive integration, sample versatility, and detection accuracy. However, it requires users to be proficient in the phone’s professional camera mode to minimize noise and adjust settings effectively. Additionally, the device could benefit from automated calibration and enhanced mechanical stability. The filters in the current camera module restrict detectable wavelengths to below 700 nm. However, this limitation could potentially be overcome by incorporating a CMOS camera and microprocessor [31].

## 4. Conclusions

In conclusion, we developed a smartphone-based spectrometer utilizing optical lenses and 3D-printed housings. This device is capable of reproducibly collecting and analyzing transmission, fluorescence, and reflectance spectra with high accuracy while being flexible, lightweight, and multifunctional. Employing a highly hydrolyzed aqueous aspirin solution, we established a simple, non-toxic, high SNR method for detecting caffeine. The system is straightforward and customizable, enabling accurate detection of caffeine in beverages. The portable spectrometer, with a small size of 120 mm × 40 mm × 107.3 mm, is cost-effective (USD 314) and facilitates the testing of food, pharmaceutical, and biological samples outside of traditional laboratory settings.

## Figures and Tables

**Table 1:** Recovery results of five diluted samples.

Sample	Dilution Factor	Add (μM)	Found (μM)	Recovery (%, n = 3)
S1	150	0 10	11.62 21.69	100.6667
S2	200	0 10	14.83 25.39	105.6000
S3	800	0 10	12.06 21.89	98.2667
S4	1000	0 10	15.98 25.78	98.0333
S5	500	0 10	26.72 36.22	105.0333

The data shown in green and red were used to calculate the original caffeine content in five samples.

## Data Availability

The data presented in this study are available on request from the corresponding author.
